# Inhibition of CCR2 attenuates neuroinflammation and neuronal apoptosis after subarachnoid hemorrhage through the PI3K/Akt pathway

**DOI:** 10.1186/s12974-022-02676-8

**Published:** 2022-12-25

**Authors:** Qi Tian, Yujia Guo, Shi Feng, Chengli Liu, Peibang He, Jianfeng Wang, Wenrui Han, Chen Yang, Zhan Zhang, Mingchang Li

**Affiliations:** 1 Department of Neurosurgery, Renmin Hospital of Wuhan University, 99 Ziyang Road, Wuhan, 430060 Hubei China; 2 Department of Rehabilitation, Renmin Hospital of Wuhan University, 99 Ziyang Road, Wuhan, 430060 Hubei China

**Keywords:** Apoptosis, CCR2, Inflammation, Neuron, Subarachnoid hemorrhage

## Abstract

**Background:**

Neuroinflammation and neuronal apoptosis are closely associated with a poor prognosis in patients with subarachnoid hemorrhage (SAH). We investigated the role of C–C motif chemokine receptor 2 (CCR2) in SAH.

**Methods:**

Pre-processed RNA-seq transcriptome datasets GSE167110 and GSE79416 from the Gene Expression Omnibus (GEO) database were screened for genes differentially expressed between mice with SAH and control mice, using bioinformatics analysis. The endovascular perforation model was performed to establish SAH. RS504393 (a CCR2 antagonist) and LY294002 (PI3K inhibitor) were administered to explore the mechanism of neuroinflammation after SAH. SAH grading, neurological scoring, brain water content and blood–brain barrier (BBB) permeability determination, enzyme-linked immunosorbent assay (ELISA), western blotting, and immunofluorescence were performed. An in vitro model of SAH was induced in H22 cells by hemin treatment. The protective mechanism of CCR2 inhibition was studied by adding RS504393 and LY294002. Clinical cerebrospinal fluid (CST) samples were detected by ELISA.

**Results:**

Expression of *CCR2* was upregulated in both datasets and was identified as a hub gene. CCR2 expression was significantly upregulated in the cytoplasm of neurons after SAH, both in vitro and in vivo. RS significantly reduced the brain water content and blood–brain barrier permeability, alleviated neuroinflammation, and reduced neuronal apoptosis after SAH. Additionally, the protective effects of CCR2 inhibition were abolished by LY treatment. Finally, the levels of CCR2, inflammatory factors, and apoptotic factors were elevated in the CSF of patients with SAH. CCR2 levels were associated with patient outcomes at the 6-month follow-up.

**Conclusion:**

CCR2 expression was upregulated in both in vitro and in vivo SAH models. Additionally, inhibition of CCR2, at least partly through the PI3K/AKT pathway, alleviated neuroinflammation and neuronal apoptosis in vivo and in vitro. CCR2 levels in the CSF have a moderate diagnostic value for 6-month outcome prediction in patients with SAH.

**Supplementary Information:**

The online version contains supplementary material available at 10.1186/s12974-022-02676-8

## Introduction

Subarachnoid hemorrhage (SAH), typically caused by a ruptured intracranial aneurysm, accounts for 5% of stroke [1]. SAH is a cerebrovascular disease with high morbidity and mortality rates. Early brain injury (EBI), occurring within 72 h, is the main cause of death and delayed neurological impairment after SAH [2]. However, the specific molecular mechanism underlying EBI after SAH remains unclear. Neuroinflammation and neuronal apoptosis are key destructive processes in SAH-induced EBI [3, 4]. Therefore, reducing neuroinflammation and neuronal apoptosis is particularly important to improve SAH prognosis.

In an asthma model, activation of the seven-transmembrane domain G-protein-coupled receptor CCR2 mediates neutrophil apoptosis through the PI3K/Akt/ERK/NF-κB pathway [5]. This receptor can be activated by CCL2, CCL7, CCL8, CCL12, CCL13, and CCL16 [6]. CCR2 is expressed in cells in different regions of the human brain, including endothelial cells, neurons, astrocytes, and microglia, in different regions of the cerebral cortex, caudate putamen, and substantia nigra, with the highest expression in the hippocampus [7, 8]. To date, studies on CCR2 in the central nervous system have mainly focused on multiple sclerosis, Alzheimer's disease, and ischemic stroke [6].

CCR2 inhibition is thought to be a therapeutic strategy for various diseases, including autoimmune diseases, atherosclerotic pain, metabolic diseases, and central nervous system diseases, partly owing to the critical role of CCR in monocyte migration and inflammation [9, 10]. RS504393 (RS) is a selective CCR2 chemokine receptor antagonist. RS was shown to significantly downregulate lipopolysaccharide (LPS)-induced *IL1β* and *PAI-1* mRNA and protein expression [11]. Additionally, RS significantly inhibited LPS-induced pulmonary edema [12] and reduced renal injury induced by renal interstitial fibrosis [13].

Therefore, we hypothesized that CCR2 is an important regulator of neuroinflammation and neuronal apoptosis induced by SAH. We used in vivo and in vitro SAH models to determine the role of CCR2 in SAH and explore the related mechanisms. Moreover, the levels of CCR2 and inflammatory and apoptotic factors in cerebrospinal fluid (CSF) samples of healthy controls and patients with SAH were detected, and the relationship between CCR2 and patient outcomes was evaluated.

## Methods

### Study design

**Fig. 1:**
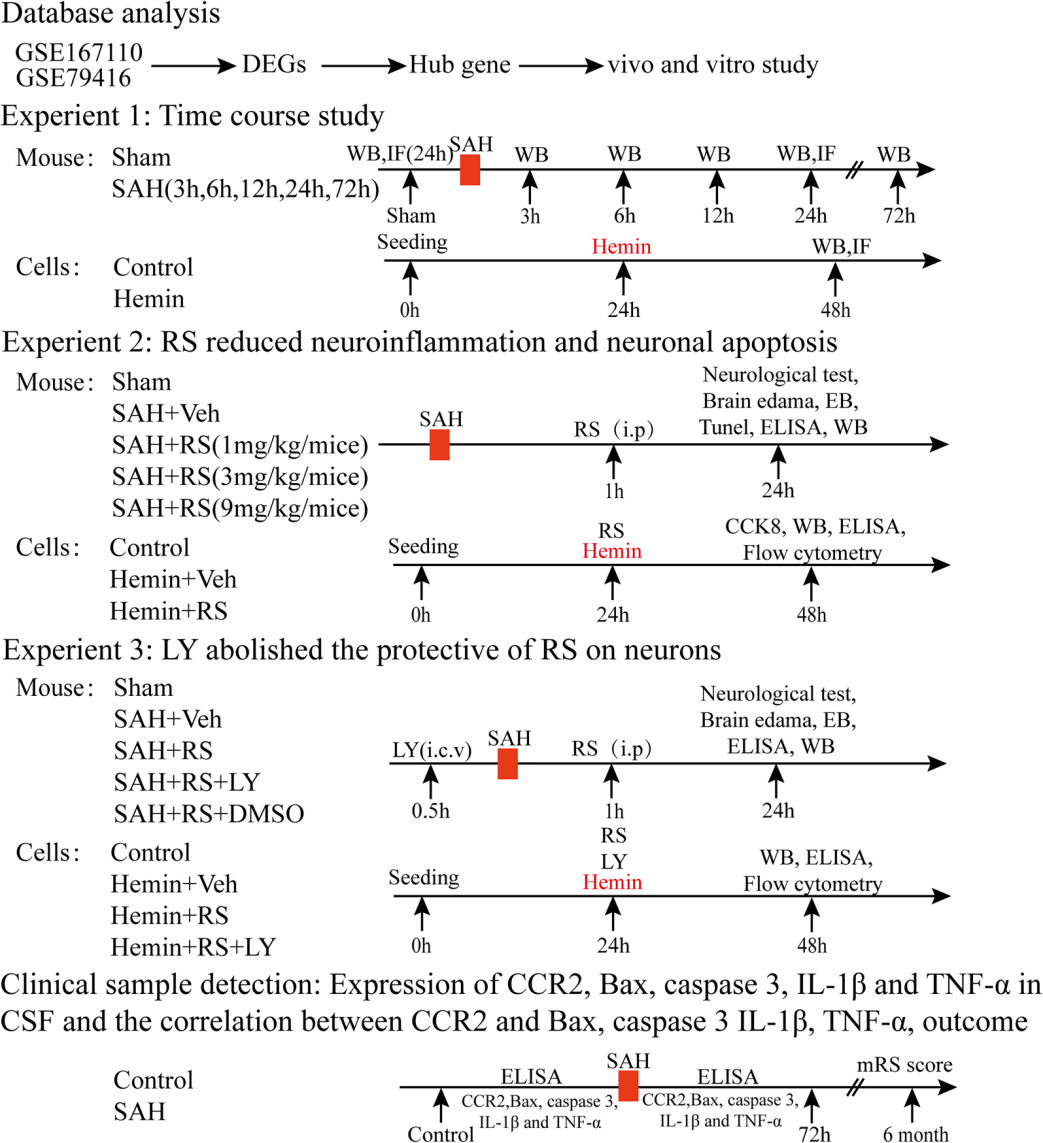
Experimental designs. RS (RS504394, CCR2 specific antagonist); LY (LY294002, PI3K specific inhibitor)

GEO Database Analysis, three separated in vitro and in vivo experiments, and clinical sample detection were conducted, as shown in Fig. 1.

### Bioinformatics analysis

The Gene Expression Omnibus (GEO, http://www.ncbi.nlm.nih.gov/geo) database was used to collect pre-processed RNA-seq transcriptome data of the brain tissues of SAH model mice, which included the GSE167110 and GSE79416 datasets. The datasets of differential RNA-seq transcriptome data in SAH were compared with those of control mouse brain tissue using the DESeq2 software package (version 1.34.0), using R software (https://www.r-project.org/) [14]. To establish statistical significance, GSE167110 was filtered using *P* < 0.05 and |fold-change (FC)|> 1 as cutoffs. GSE79416 was filtered using *P* < 0.05 and |FC|> 2 cutoffs. A Venn diagram was constructed to identify the intersecting differentially expressed genes (DEGs) using the Venn package (version 1.10) in R. A protein–protein interaction (PPI) network of intersecting DEGs was visualized using the STRING database (https://string-db.org/) and Cytoscape software (version 3.9.1) [15]. CytoHubba in the Cytoscape software was used to identify the top-10 hub genes and key modules of the PPI network [16]. GeneMANIA web server [17] (http://www.genemania.org/) was used to identify the gene interaction networks. Gene Ontology (GO) enrichment and Kyoto Encyclopedia of Genes and Genomes (KEGG) pathway analyses were conducted using the package cluster profiler (version 4.4.2) [18] of R (version 4.1.0) software, which showed the biological processes, cellular components, molecular functions, and pathways related to the intersecting DEGs. Only terms with *P* < 0.05 were considered statistically significantly enriched pathways. The ggplot2 R package was used to visualize the results.

### Animals

All animal experiments were approved by the Animal Experiment Center of Wuhan University (WDRM—20170504). Wild-type C57BL/6J male mice (total of 261 mice including 221 SAH mice and 40 Sham mice, 25–30 g) were purchased from the Central Laboratory of Renmin Hospital of Wuhan University. Before animal experiments, the animals were acclimated in a room with controlled humidity (65 ± 5%) and temperature (25 ± 1 °C), with a 12 h/12 h light/dark cycle and with free access to food and water for at least 1 week.

### SAH model

Intravascular perforations were performed as previously described [19]. Mice were anesthetized with 5% isoflurane and maintained with 2% isoflurane. The mice were placed in the supine position, and the skin was incised in the middle of the neck to separate the common carotid, external carotid, and internal carotid arteries. A 4-0 monofilament was then inserted from the left external carotid artery to the internal carotid artery and continued forward until resistance was encountered to puncture the vessel. The sutures were rapidly removed, the external carotid artery ligated, and the wound sutured. A sham group of mice underwent a similar surgery but without perforation.

### Drug administration

The mice were administered RS (SML0711-5MG, Sigma, USA) at different dosages (1, 3, and 9 mg/kg/day, intraperitoneally). According to the weight of the mice, an appropriate amount of RS was weighed and dissolved in 5 ul phosphate-buffered saline [PBS, containing 10% dimethyl sulfoxide (DMSO)] and intraperitoneally injected after SAH induction for 1 h. Similarly, 5 μL of PBS containing 10% DMSO was used as the negative control for RS. According to previous studies, LY294002 (LY, #S1737-5 mg, Beyotime, Shanghai, China) was prepared in 50 mmol/L PBS containing 10% DMSO with a total volume of 5 μL and was injected in the cerebral ventricle (i.c.v) 30 min before SAH induction [20]. Similarly, 5 μL of PBS containing 10% DMSO was used as the negative control for LY.

### SAH grading

The SAH grading score was determined by an independent investigator blinded to the experimental group at 24 h after SAH was induced, as previously described [21]. SAH mice with a score of ≤ 8 were excluded from the study.

### Neurological function evaluation

Neurological performance was evaluated 24 h after SAH induction in a blinded fashion, using a modified Garcia scale, as previously described [22]. The modified Garcia test (scores of 3–18) included whisker contact, trunk contact, spontaneous movement, spontaneous limb movement, forelimb extension, and climbing ability.

### Brain water content

Brain tissue was collected at 24 h after SAH induction and was divided into left and right hemispheres. The brain tissue was weighed immediately to obtain the wet weight and was then weighed after drying at 105 ℃ for 24 h (dry weight). The percentage of brain water content was calculated as follows: [(wet weight − dry weight)/wet weight] × 100%.

### Blood–brain barrier permeability

The blood–brain barrier (BBB) permeability was assessed 24 h after SAH induction, as described in a previous study [23]. Briefly, Evans blue (EB, 2%, 5 mL/kg, Sigma) was injected into the left ventricle 1 h before killing. After killing, the brain tissue was homogenized in 50% trichloroacetic acid (TCA), after which the samples were centrifuged for 5 min at 1000×*g*. The supernatant was collected and mixed with ethanol and TCA at 4 ℃ overnight. EB concentration was determined by measuring the absorbance at 630 nm, which represented BBB permeability.

### Immunofluorescence staining

Double-immunofluorescence staining was performed as described in a previous study [24]. The mice were deeply anesthetized 24 h after SAH induction, and 60 mL of frozen PBS was administered via the heart, followed by 60 mL of 4% paraformaldehyde. The whole brain was removed, fixed with 4% paraformaldehyde for 24 h, paraffin-embedded, and sectioned. To perform double-immunohistochemical staining, the brain tissue sections were incubated with primary antibodies mouse anti-NeuN (#94403, 1:200, Cell Signaling Technology, CST) and rabbit anti-CCR2 (#ab216863, 1:200, Abcam) overnight at 4 ℃. The sections were incubated with the appropriate secondary antibody at room temperature for 1 h. The sections were then visualized under a fluorescence microscope and photographed. Six coronal sections showing the ipsilateral cortex and hippocampus were randomly selected. CCR2- and TNF-α-positive cells were detected and counted in three different regions. The data are expressed as the number of cells/fields.

### TUNEL staining

Double staining was performed for the neuronal marker NeuN and terminal deoxynucleotide transferase dUTP notched-end labeling (TUNEL) marker, using an apoptosis detection kit (#G1501-50T, Servicebio, Wuhan, China) according to the manufacturer’s instructions, in brain tissues obtained 24 h after SAH induction. The number of TUNEL-positive neurons in the ipsilateral cortex and hippocampus was manually counted under 40× magnification. An average of six slices were investigated for each brain region. Data are expressed as the number of TUNEL-positive neurons (%).

### Cell culture and in vitro SAH model

For the in vitro study, murine HT22 cells were cultured in Dulbecco's modified Eagle medium (‘DEME’) containing 10% fetal bovine serum and 1% penicillin/streptomycin in a humidified atmosphere containing 5% carbon dioxide at 37 ℃. Hemin (160 μM) was introduced into the culture medium for 24 h to induce SAH damage in vitro according to the cell counting kit-8 (CCK-8) assay (#G4103-5ML, Servicebio, Wuhan, China) results [19, 25]. After incubation, the medium was removed, and the cells were washed with PBS. HT22 cells were pretreated with 10 μM RS and 10 μM LY for 1 h to observe the mechanism of CCR2 after SAH.

### Cell viability

Cell viability was assessed using a CCK-8 kit. HT22 cells were seeded in 96-well plates. SAH was induced as described above. CCK-8 reagent (10 µL) was mixed with 90 µL of complete medium and added to each well, and the plates were incubated for 1 h. The optical density was measured at 450 nm using a microplate reader. The results are expressed as the percentage of living cells, and the cell survival rate of the control group was set as 100%.

### Flow cytometry

Flow cytometry was performed to assess neuronal death using an Annexin V PE/7-AAD kit (#CA1030, SolarBio, Beijing, China). The data were analyzed using Novocyte Express (Santa Clara, CA, USA). Dead cell count was calculated using the following formula: [number of Annexin V PE+/7-AAD+ cells/number of total cells] × 100%.

### Western blotting

Western blotting was performed as previously described [26]. Proteins from the brain samples and cultured HT22 cells were lysed with RIPA lysis buffer. Equal amounts of protein (50 μg) were loaded onto a sodium dodecyl sulfate–polyacrylamide gel. The proteins were electrophoresed until they were fully separated and then transferred to a polyvinylidene difluoride membrane. The membrane was then blocked with 5% bovine serum albumin for 1 h at room temperature. The membranes were incubated with the following primary antibodies overnight at 4 ℃: anti-CCR2 (#12199, 1:1000, CST), anti-p-PI3K (#4228, 1:1000, CST), anti-PI3K (#AF7742, 1:1000, Beyotime), anti-AKT (#4691, 1:1000, CST), anti-pAKT (#4060, 1:1000, CST), anti-IL-1β (#ab254360, 1:1000, Abcam), anti-TNF-α (#ab255275, 1:1000, Abcam), anti-BAX (#GB114122, 1:1000, ServiceBio), anti-Bcl-2 (#GB113375, 1:1000, ServiceBio), anti-cleaved caspase 3 (#ab2302, CC3, 1:1000, Abcam), and anti-β-tubulin (#GB11017, 1:1000, ServiceBio). Suitable secondary antibodies (1:5000, Santa Cruz Biotechnology, Dallas, TX, USA) were selected and incubated with the membrane at room temperature for 1 h. The bands were then observed using an enhanced chemiluminescence reagent. Image J software (NIH, Bethesda, MD, USA) was used for density measurements and quantification.

### Participants

The study was approved by the ethics committee of our institution. All participants provided written informed consent. Patients with aneurysm SAH (aSAH) were recruited at our hospital between January 2019 and April 2022. Cerebral computed tomography showed a SAH, and digital subtraction angiography (DSA) or computed tomography angiography (CTA) suggested an intracranial aneurysm. The inclusion criteria for aSAH were: (1) age between 18 and 75 years, (2) aSAH confirmed by cerebral DSA and/or CTA and CT, (3) CSF collected within 3 days after aSAH, and (4) informed consent signed by the participant's next of kin. Healthy individuals were recruited as controls. The inclusion criteria for the control group were as follows: (1) age between 18 and 75 years; (2) no current or prior brain injury, neurological disease, or bleeding disorder; and (3) the participant or a close relative signed a consent form. The modified Rankin Scale (mRS, 0–6 score) was recorded at the 6-month follow-up. For analysis in this study, we collapsed the mRS scores to 0–2 (favorable outcome) vs 3–6 (unfavorable outcome) [27]. The control group CSF was obtained from patients without intracranial or spinal lesions who required spinal anesthesia for other reasons. The samples were centrifuged at 4 ℃ at 2500×*g* for 10 min to remove erythrocytes and were stored at − 80 °C until analysis.

### ELISA detection

Mouse IL-1β (MU30369, Bioswamp, Wuhan, China) and TNF-α (MU30030, Bioswamp, Wuhan, China) levels were tested using an ELISA kit, according to the manufacturer's instructions, to investigate the effect of CCR2 on inflammatory changes. CCR2 (HM11536, Bioswamp, Wuhan, China), BAX (HM10117, Bioswamp, Wuhan, China), caspase 3 (HM10963, Bioswamp, Wuhan, China), IL-1β (HM10206, Bioswamp, Wuhan, China), and TNF-α (HM10001, Bioswamp, Wuhan, China) levels were analyzed in human CSF using ELISA. The optimal dilution was determined, and ELISA was performed according to the manufacturer’s instructions.

### Statistical analysis

RNA-seq data were statistically analyzed using R Studio (version 4.1.0; https://www.r-project.org/). GraphPad Prism software (version 8.02; GraphPad Inc., La Jolla, CA, USA) was used for the statistical analysis. All data were expressed as mean ± standard deviation. Student’s t-test was used for comparison between two groups. One-way analysis of variance (ANOVA) was performed if the comparison involved three groups and the group above. Pearson’s correlation test was used to evaluate whether there was a correlation between CCR2 and BAX, caspase 3, IL-1β, TNF-α, and mRS scores. The area under the ROC curve and 95% confidence interval (CI) were determined. The sensitivity and specificity of each independent risk factor were obtained for a range of different cut-off points. *P* < 0.05 was considered statistically significant.

## Results

### Bioinformatics analysis

**Fig. 2:**
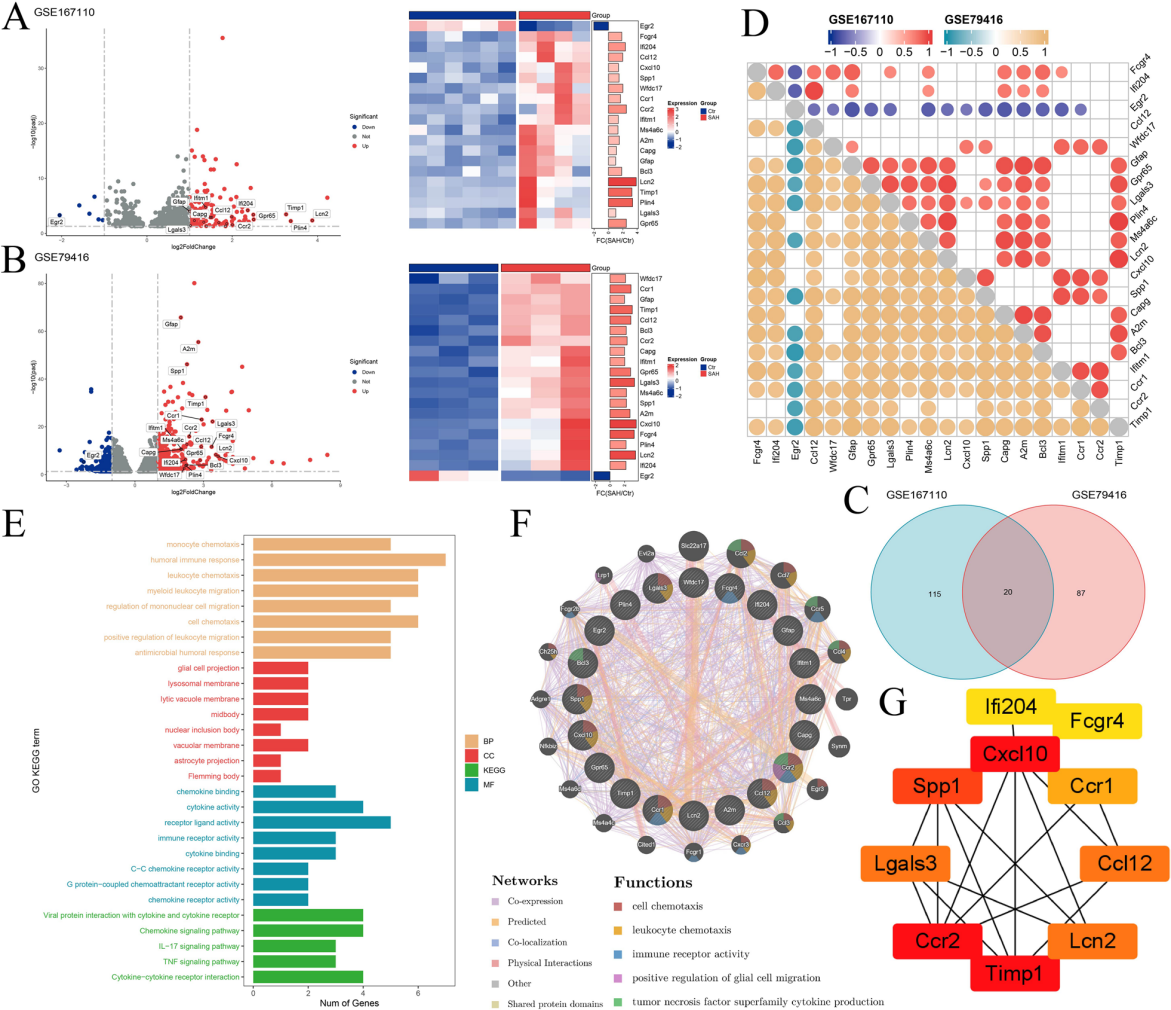
Bioinformatics analysis of DEGs between SAH and control. **A**, **B** Volcano plots and heatmaps of the intersected DEGs expression levels in the **A** GSE167110 and **B** GSE79416. **C** Venn diagram showing DEGs in the three datasets. **D** Correlations among the intersected DEGs between SAH and control in the two datasets via Pearson analysis. Red represented positive correlation and blue represented negative correlation in GSE167110; orange represented positive correlation and green represented negative correlation in GSE79416. **E** Enriched items in GO and KEGG analysis. GO: Gene Ontology; BP: biological process; CC: cellular component; MF: molecular function; KEGG: Kyoto Encyclopedia of Genes and Genomes. **F** PPI network of the intersected DEGs in the GeneMINIA. **G** The Cytohubba was used to construct the Top10 hub genes. The figure showed the Top10 hub genes constructed by the NHC method

We identified 135 DEGs between SAH and control samples in GSE167110, including 129 upregulated and six downregulated genes. Based on data from three mice with SAH and three normal controls in GSE79416, we identified 107 DEGs, of which 95 were upregulated and 12 were downregulated. By investigating intersecting DEGs in GSE167110 and GSE79416 by Venn diagram analysis, we identified 20 DEGs for further analyses, of which 19 were upregulated and one was downregulated (Fig. 1C). These intersecting DEGs included *CCR2*. The expression of intersected DEGs in each GEO dataset is presented as a volcano plot and heatmap (Fig. 2A, B). We also analyzed the correlation of expression among the intersecting DEGs in both datasets and found that most intersecting DEGs were positively correlated with each other (Fig. 2D).

To discover the common transcription-level changes in SAH and reveal the role of the functional pathways, we performed GO functional and KEGG pathway enrichment analyses of the intersecting DEGs and ranked enriched terms based on the number of intersecting genes involved. GO analysis showed that the DEGs were most significantly enriched in the biological activity of immune cells, such as humoral immune response, leukocyte chemotaxis, myeloid leukocyte migration, receptor ligand activity, cytokine activity, and immune receptor activity. KEGG analysis showed that intersecting DEGs were mainly associated with immune cell-related signaling pathways, such as the chemokine signaling pathway, cytokine–cytokine receptor interaction, the IL-17 signaling pathway, and the TNF signaling pathway (Fig. 2E).

We predicted the PPI network of the intersecting DEGs using GeneMANIA (Fig. 2F) and STRING tools, followed by analysis using Cytoscape software. The top-10 hub genes were identified based on the CytoHubba NHC algorithm using the Cytoscape software (Fig. 2G): *CCR2, CXCL10, TIMP1, SPP1, Lcn2, Lgals3, CCL12, CCR1, FCGR4,* and *IFI204*. *CCR2* was the upregulated gene with the highest score in the NHC method and was identified as the key hub gene of the predicted network.

### SAH mortality, grading, and exclusion

Forty mice were included in the sham group, while 271 mice were used to establish the SAH model. Of the latter, 61 mice died within 24 h, 4 mice died within 72 h, and 20 mice were excluded owing to SAH grading score < 8. The overall mortality rate in the SAH model group was 24.0% (65/271) (Additional file 1: Fig. S1A). A schematic diagram of the SAH model is shown in Additional file 1: Fig. S1B. There were no significant differences in SAH grading scores among all SAH groups (Additional file 1: Fig. S1C).

### Temporal expression and localization of CCR2 after SAH induction in vitro and in vivo

**Fig. 3:**
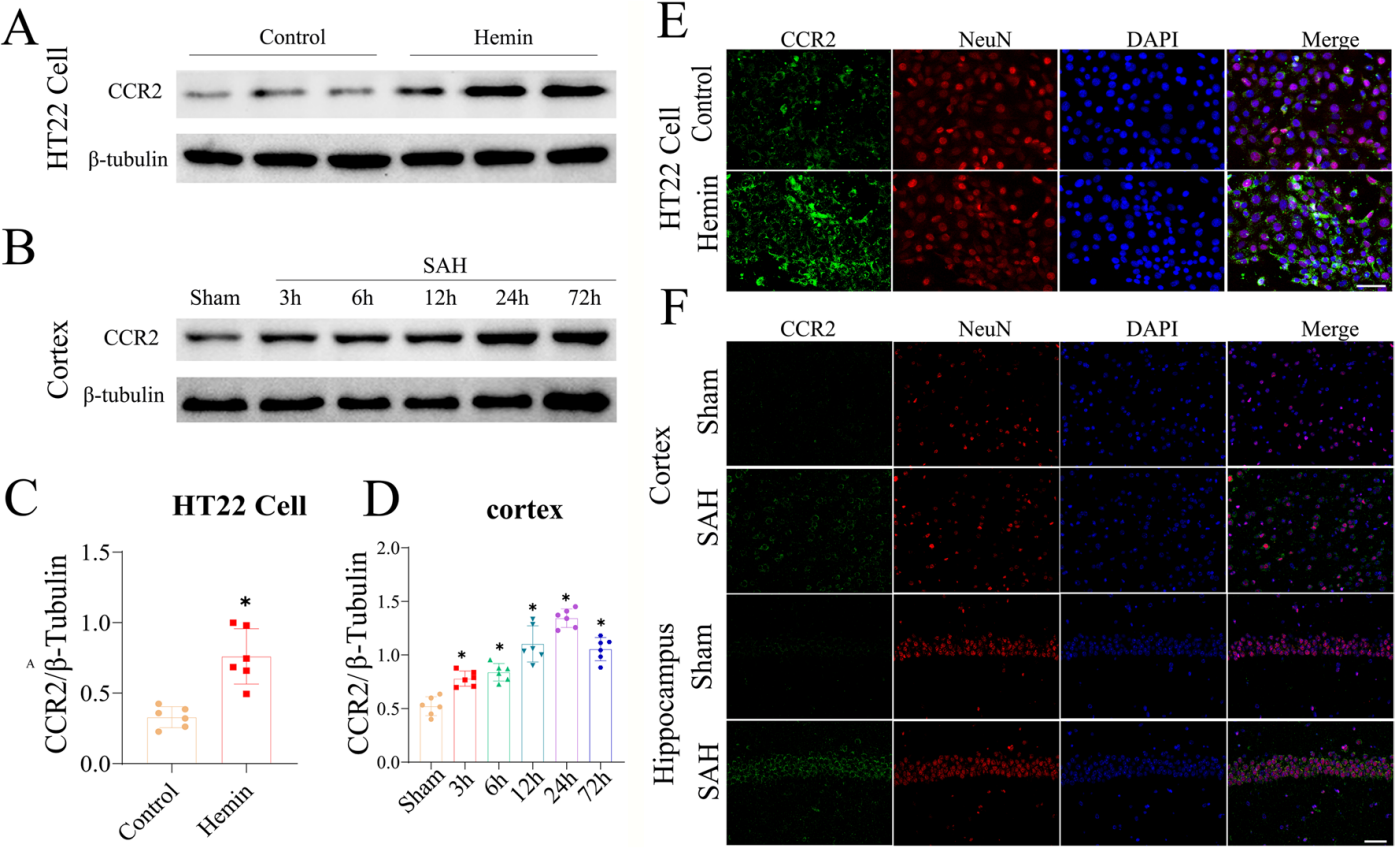
Time course of CCR2 expressions after SAH in vitro and vivo. Representative western blots bands of time course and densitometric quantification of CCR2 in vitro (**A** and** C**) and vivo (**B** and **D**). Colocalization of CCR2 with neuron in HT22 (**E**), cortex and hippocampus tissues (**F**) at 24 h after hemin induced or operator SAH. Scale bar = 50 µm, *n* = 6 per group. *P < 0.05 vs Sham group. Scale bars = 50 μm

In vitro study, western blotting and immunofluorescence showed that the CCR2 level was upregulated in the hemin-induced SAH model after 24 h and was expressed in the cytoplasm (Fig. 3A, C,  E). Western blotting and immunofluorescence showed that CCR2 protein levels gradually increased by 3 h after SAH, peaked at 24 h after SAH, and gradually decreased up to 72 h after SAH (Fig. 3B, D and F).

In the in vivo study, CCR2 was expressed in the membrane and cytoplasm of neurons, and the expression of CCR2 was higher in the cortex and hippocampus tissues in the SAH group than in the sham group (Fig. 3D).

### RS alleviates hemin-induced neuroinflammation and neuronal apoptosis in vitro

**Fig. 4:**
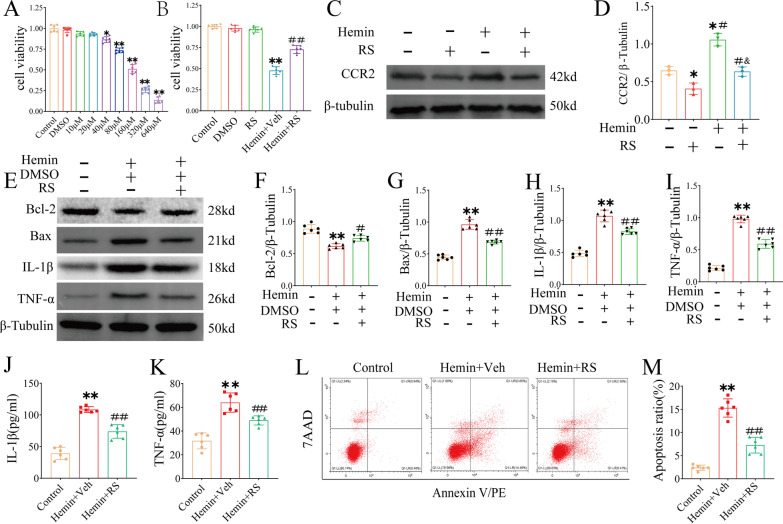
CCR2 increased cell viability and inhibited hemin-induced cell inflammation and apoptosis at 24 h after hemin exposure. **A** Cell viability decreased with increasing hemin concentration (*n* = 6). **B** CCK-8 results showed that the cell viability decreased significantly after treatment with hemin (160 mmol/L). RS (10 μmol/L; *n* = 6) rescued the process. **C**, **D** Representative western blot strips and CCR2 density quantified the efficacy of RS on hemin-induced SAH, *n* = 3 per group. **E**–**I** Representative western blot bands and densitometric quantification of Blc-2, Bax, IL-1β and TNF-α in hemin-induced SAH in vitro, *n* = 6 per group. **J**, **K** ELISA detected the concentrations of IL-1β and TNF-α in cell lysates after homogenization. **L**, **M** Representative flow cytometry images of HT22 cells, apoptotic cells represented by 7-AAD+/Annexin V+ ratio. *P < 0.05 vs Sham group. **P < 0.01 vs Sham group. ^#^*P* < 0.05 vs SAH group. ^##^*P* < 0.01 vs SAH group

Hemin-induced HT22 cell damage was used to establish an in vitro SAH model. HT22 cells were exposed to different concentrations of hemin (10–640 μmol/L) for 24 h, and cell viability was assessed using the CCK-8 assay (Fig. 4A). The results showed that hemin induced cell death in a concentration-dependent manner.

Next, 160 μmol/L hemin (resulting in cell viability 51.12 ± 5.31%) was used to evaluate the protective effect of RS in vitro. Western blotting results showed that CCR2 levels were decreased in both the control and hemin groups after RS treatment (Fig. 4C, D). Moreover, the reduction in cell viability induced by hemin was significantly reversed by RS treatment (Fig. 4B). Furthermore, western blotting results showed that RS downregulated expression of BAX, IL-1β, and TNF-α and upregulated expression of BCL-2 as compared to those in the hemin group (Fig. 4E–I). ELISA results showed that IL-1β (Fig. 4J) and TNF-α (Fig. 4K) levels were lower in the RS-treated group than in the hemin group. Flow cytometry confirmed that RS reduced hemin-induced apoptosis (Fig. 4L, M).

### RS improves short-term neurobehavioral deficits and reduces brain edema and BBB permeability at 24 h and 72 h after SAH induction

**Fig. 5:**
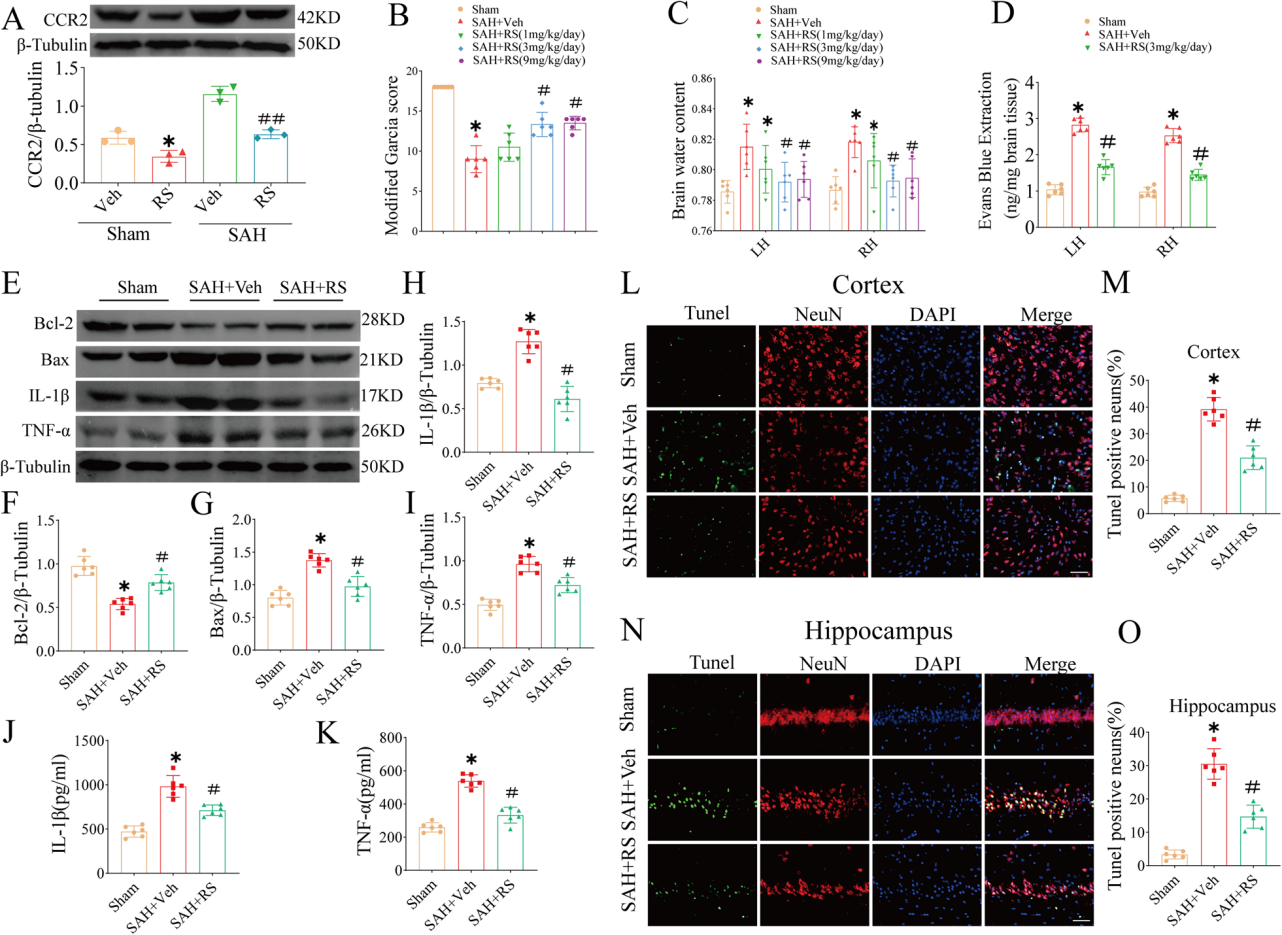
CCR2 attenuated the neuroinflammation and neuronal apoptosis after SAH. **A** The expression of CCR2 was significantly reduced in the ipsilateral cortex by RS at 24 h after SAH. **B**–**D** Inhibition of CCR2 using RS improves neurological and reduces brain edema and EB extravasation at 24 h following SAH. **E**–**I** Representative western blot bands and densitometric quantification of Blc-2, and Bax, IL-1β, TNF-α in the ipsilateral hemisphere at 24 h after SAH, *n* = 6 per group. **J**, **K** The concentrations of IL-1β and TNF-α in ipsilateral cortical tissue after homogenization were determined by ELISA. **L**–**O** Representative images of TUNEL staining and quantification of TUNEL-positive neurons in cortex and hippocampus tissues. Veh, PBS containing 10% DMSO. *P < 0.05 vs Sham group. **P < 0.01 vs Sham group. ^#^*P* < 0.05 vs SAH group. ^##^*P* < 0.01 vs SAH group. Scale bars = 50 μm

To verify the protective effect of RS after SAH, we intraperitoneally injected RS into SAH and sham mice to inhibit CCR2. Western blotting results showed that RS downregulated CCR2 expression by 24 h after injection (Fig. 5A). The neurobehavioral performance of SAH animals was worse, and the brain water content was significantly higher than that of sham animals at 24 h after SAH. RS significantly increased the modified Garcia score and reduced cerebral edema, as compared with those of untreated SAH animals, at doses of 3 and 9 mg/kg/day (Fig. 5B, C). Based on the short-term neurobehavioral function and brain water content, a dose of 3 mg/kg was selected for the rest of the experiment. BBB permeability was evaluated based on EB extravasation in both hemispheres. EB extravasation was increased significantly at 24 h after SAH, while RS treatment (3 mg/kg/day) significantly reduced EB dye leakage in both hemispheres (Fig. 5C). In addition, similar to the 24-h effect, we evaluated the results of RS treatment at 72 h after SAH and found that the RS group had improved neural function scores, decreased cerebral water content, and reduced blood–brain barrier destruction compared to the SAH group (Additional file 2: Fig. S2A–C).

### RS reduces neuroinflammation and neuronal apoptosis at 24 h after SAH induction

Compared with that in the sham operation group, the expression of BAX, IL-1β, and TNF-α in the SAH + vehicle (SAH + Veh) group was significantly upregulated, and the expression of BCL-2 was significantly downregulated. The pharmacological action of RS against CCR2 significantly downregulated the expression of BAX, IL-1β, and TNF-α and upregulated the expression of BCL-2 (Fig. 5E–I). In addition, ELISA was used to detect the levels of IL-1β and TNF, and the results showed that IL-1β and TNF-α levels were increased after SAH but were decreased after RS treatment (Fig. 5J, K).

TUNEL staining was used to evaluate neuronal apoptosis. The results showed that the number of apoptotic neurons in the cortex and hippocampus of the SAH + Veh group was increased significantly at 24 h after SAH (*P* < 0.05, Fig. 5L–M). In contrast, RS treatment inhibited neuronal apoptosis in the cortex and hippocampus (*P* < 0.05, Fig. 5L–M).

### LY abolishes the anti-inflammatory and anti-neuronal apoptosis effects of RS in vitro

**Fig. 6:**
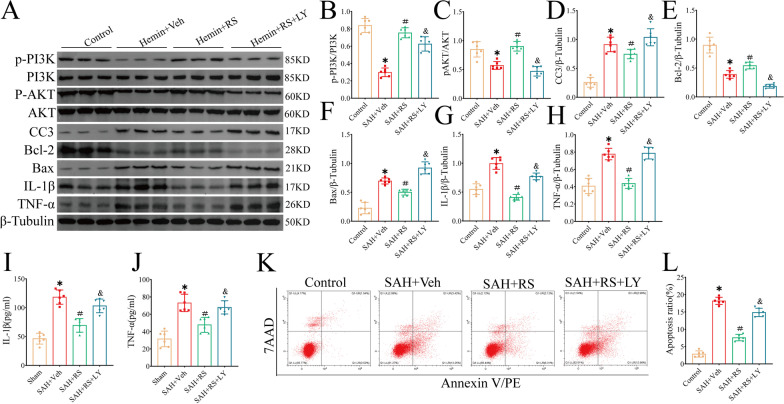
LY abolished the anti-neuroinflammation and anti-apoptosis effects of RS at 24 h in vitro. **A** Representative western blot bands. **B**–**H** Densitometric quantification of CCR2, p-PI3K, PI3K, p-AKT, AKT, Bcl-2, Bax, and CC3, IL-1β and TNF-α at 24 after hemin-induced SAH. **I**, **J** ELISA detected the concentrations of IL-1β and TNF-α in cell lysates after homogenization. **K**, **L** Representative flow cytometry images of HT22 cells, apoptotic cells represented by 7-AAD+/Annexin V+ ratio. *P < 0.05 vs Sham group; **P < 0.01 vs Sham group. #*P* < 0.05 vs Hemin group. ^##^*P* < 0.01 vs Hemin group; ^&^*P* < 0.05 vs. Hemin + RS group; ^&&^*P* < 0.01 vs. Hemin + RS group; *n* = 6 per group

Western blotting further confirmed that RS upregulated the expression of p-PI3K, p-AKT, and BCL-2, while it downregulated the expression of pro-inflammatory mediators (IL-1β and TNF-α) and pro-apoptotic mediators (CC3 and BAX) in vitro. LY reversed the effects of RS (Fig. 6A–H). ELISA results showed that IL-1β (Fig. 6I) and TNF-α (Fig. 6J) levels in the hemin + RS + LY group were significantly higher than those in the hemin + RS group. Similarly, flow cytometry showed that RS reduced hemin-induced apoptosis, whereas LY reversed the effect of RS and increased apoptosis (Fig. 6K, L).

### LY reverses the protective effects of RS at 24 h after SAH induction

**Fig. 7:**
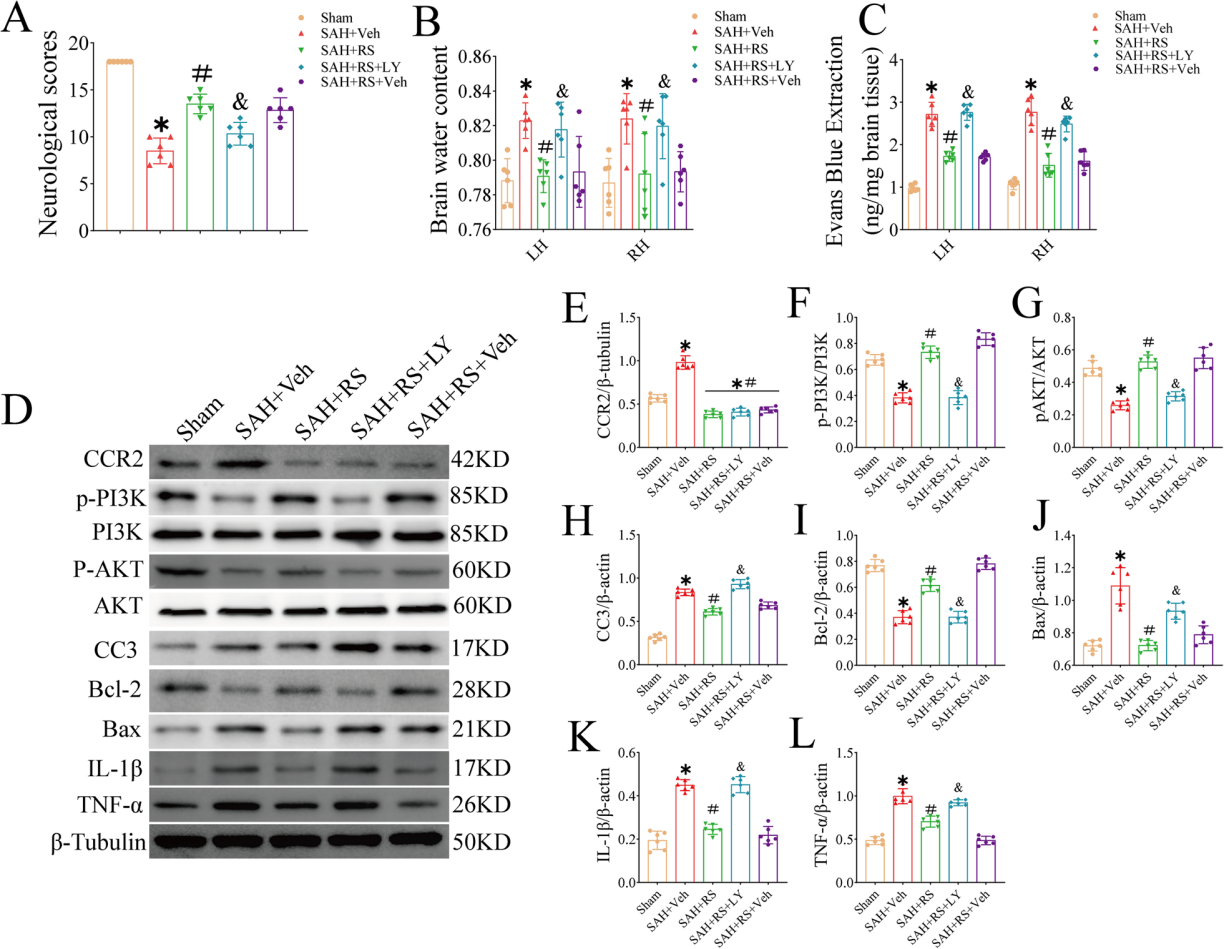
LY reversed the protective effects of RS at 24 h after SAH in mice. **A**–**C** LY aggravates neurological and increases brain edema and EB extravasation at 24 h following SAH treated with RS. **D** Representative western blot bands. **E**–**L** Densitometric quantification of CCR2, p-PI3K, PI3K, p-AKT, AKT, Bcl-2, Bax, and CC3, IL-1β and TNF-α in the ipsilateral hemisphere at 24 h after SAH. Data were represented as mean ± SD. Veh, PBS containing 10% DMSO. *P < 0.05 vs Sham group; **P < 0.01 vs Sham group. ^#^*P* < 0.05 vs SAH group. ^##^*P* < 0.01 vs SAH group; ^&^*P* < 0.05 vs. SAH + RS and SAH + RS + DMSO group; ^&&^*P* < 0.01 vs. SAH + RS and SAH + RS + DMSO group; *n* = 6 per group

Intraventricular injection of LY inhibited the PI3K pathway. Inhibition of the PI3K signaling pathway exacerbated neurological deficits (Fig. 7A) and increased cerebral edema (Fig. 7B) and BBB permeability (Fig. 7C) by 24 h after SAH. In addition, LY pretreatment significantly downregulated the expression of p-PI3K, p-AKT, and BCL-2 and upregulated the expression of BAX, cleaved caspase 3, IL-1β, and TNF-α (Fig. 7D–L).

### Expression of CCR2, BAX, caspase 3, IL-1β, and TNF-α in CSF and the correlation between CCR2 and BAX, caspase 3, IL-1β, and TNF-α levels and outcomes

**Fig. 8:**
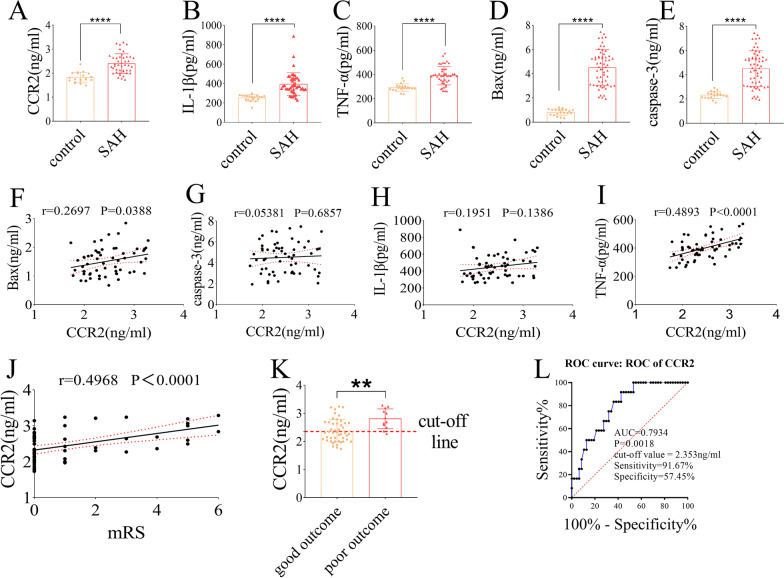
Expression of CCR2, Bax, caspase 3, IL-1β and TNF-α in CSF and the correlation between CCR2 and Bax, caspase 3 IL-1β, TNF-α, outcome. **A**–**E** The level of CCR2, Bax, caspase 3, IL-1β and TNF-α in CSF were detected by ELISA in control and SAH patients. **F**–**J** The relationship between CCR2 and Bax, caspase 3, IL-1β, TNF-α and mRS scores. **K**, **L** ROC showed that CSF CCR2 levels after SAH had a moderate diagnostic value for outcome at 6-month follow-up. Meanwhile, the cut-off value of CCR2 was 2.353 ng/mL according to the ROC curve

To clarify the changes in the levels of CCR2, inflammatory factors (IL-1β and TNF-α), and apoptotic factors (BAX and caspase 3) in the CSF of patients with SAH, we performed ELISA testing on the CSF of patients with SAH (*n* = 59) and healthy controls (*n* = 20). The results showed that the levels of CCR2, IL-1β, TNF-α, BAX, and caspase 3 in the CSF of the SAH group were higher than those in the CSF of the control group (*P* < 0.0001) (Fig. 8A–E). Pearson’s correlation coefficient was used to analyze the relationship between CCR2 levels in the CSF and BAX, caspase 3, IL-1β, and TNF-α levels and mRS scores at 6 months after discharge. The results showed that CCR2 was positively correlated with TNF-α (*r* = 0.4893, *P* < 0.001) (Fig. 8I), BAX (*r* = 0.2697, *P* = 0.0388) (Fig. 8F), and mRS score (*r* = 0.4968, *P* < 0.001) (Fig. 8J) but not with IL-1β (*r* = 0.0538, *P* = 0.6857) (Fig. 8H) and caspase 3 (*r* = 0.1951, *P* = 0.1386) (Fig. 8G). Receiver operating characteristic (ROC) curve analysis showed that CSF CCR2 levels (area under the ROC curve = 0.7934, sensitivity = 91.67%, specificity = 57.45%, *P* = 0.0018) (Fig. 8L) after SAH had a moderate diagnostic value for outcomes at the 6-month follow-up. The cut-off value of CCR2 was 2.353 ng/mL according to the ROC curve (Fig. 8K).

## Discussion

**Fig. 9:**
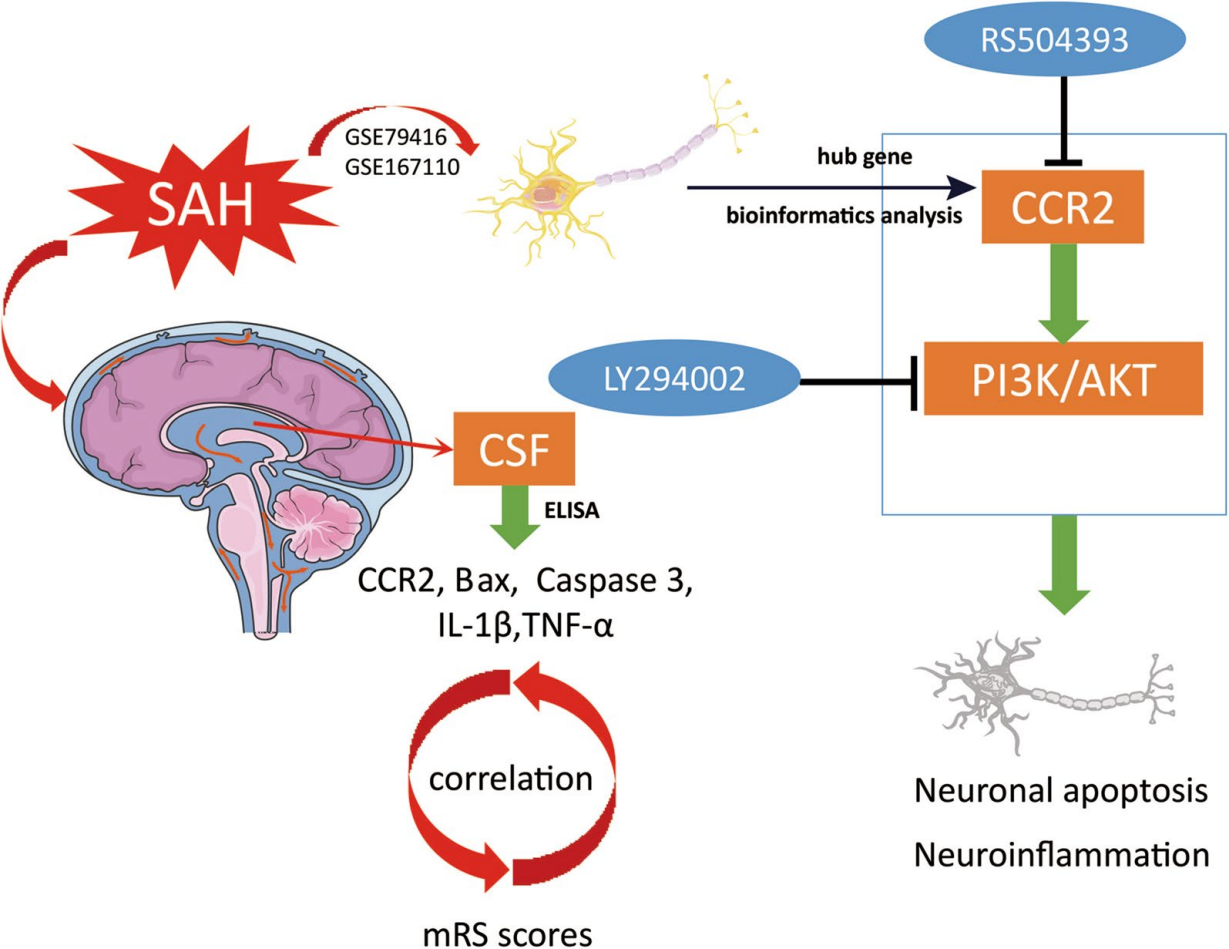
Schematic diagram for CCR2 following SAH. CCR2 was received by bioinformatics analysis after SAH. It induces early brain injury by regulating neuroinflammation and neuronal apoptosis and is positively correlated with clinical outcome of SAH patients

This study revealed the neuronal effects of CCR2 on SAH and the underlying mechanism, which have not been reported previously. In GSE167110 and GSE79416 analyses, we found that *CCR2*, as a hub gene, was highly expressed in cortical and hippocampal tissues in the SAH group, as compared with the sham group. This result was consistent with our in vitro and in vivo study results. We also demonstrated that the CCR2 antagonist RS could ameliorate neurological deficits in mice at 24 h after SAH, alleviate cerebral edema and damage to the BBB, and reduce neuroinflammation and neuronal apoptosis. Moreover, we showed that the neuroprotective effect of the CCR2 antagonist RS acted at least partly through the PI3K/AKT signaling pathway. The PI3K antagonist LY reversed the neuroprotective effects of the CCR2 antagonist RS in vitro and in vivo. Finally, we found that the level of CCR2 in CSF of SAH patients was higher than that of the control group, and was positively correlated with the mRS scores of patients at 6 months of follow-up(Fig. 9).

Neuroinflammation and neuronal apoptosis in the cerebral cortex and hippocampus are the main causes of neurological dysfunction in EBI after SAH [28–31]. In mice, CCR2 is mainly expressed in a subset of monocytes that express Ly6C at high levels [32]. *CCR2* mRNA has been detected in T cells, immature B cells, natural killer cells, basophils, and dendritic cells [6]. In the central nervous system, CCR2 is widely expressed in neurons, endothelial cells, astrocytes, and microglia in different regions of the human brain, with the highest expression in the hippocampus [6]. In our study, CCR2 levels were significantly increased in the ipsilateral cerebral hemisphere after SAH and peaked at 24 h after injury. Double-immunofluorescence staining showed that CCR2 was expressed in cortical and hippocampal neurons, as well as in HT22 cells, and that its expression was increased by 24 h after SAH induction.

Recently, CCR2 has been shown to be an important contributor to inflammation and apoptosis [6, 33]. In experimental autoimmune encephalomyelitis (EAE), a common animal model of multiple sclerosis, CCR2 expression is increased at both the onset and peak of the disease and is associated with prognosis. However, *CCR2*−/− mice had fewer infiltrating T cells and F4/80^+^ macrophages and were resistant to acute EAE [6]. Moreover, CCR2 is involved in inflammatory responses that damage the brain tissue after cerebral ischemia. Compared with that in wild-type mice, *CCR2*-deficient mice had a small cerebral infarction size after ischemia–reperfusion, which reduced BBB permeability as well as inflammatory cytokine expression [34]. There is also evidence that infiltrated CCR2^+^ Ly6Chi monocytes play a detrimental role in functional outcomes after cerebral hemorrhage [6]. After amyloid β (Aβ) injection of the retina, the most upregulated gene was the chemokine gene *CCL2*, whose deletion or that of its homologous receptor *CCR2* greatly reduced the migration of activated microglia to the site of retinal injury, confirming that Aβ and CCL2/CCR2 could promote inflammation and apoptosis [35]. In addition, ethanol has been found to induce microglial activation and neuroinflammation as well as a sharp increase in *CCR2* mRNA and protein levels. Treatment with the CCR2 synthesis inhibitor RS significantly reduced microglial activation, neuroinflammation, and neuronal apoptosis in the brain [36]. However, there have been few studies on CCR2 in SAH, and these studies have mainly focused on the expression and mechanism of CCR2 in microglia after SAH [37–39]. The relationship among CCR2, neuroinflammation, and neuronal apoptosis after SAH has not been reported. We showed that CCR2 expression was upregulated in neurons after SAH and that it promoted neuroinflammation and neuronal apoptosis.

Some researchers have evaluated CCR2 as a therapeutic target based on its important role in various diseases. Many CCR2 antagonists have also emerged [9, 40]. A phase IIa clinical trial of a human CCR2 antagonist (MLN1202) in patients with active rheumatoid arthritis found that treatment with the CCR2 antagonist reduced free CCR2 levels on CD14^+^ monocytes by at least 57% and up to 94%. However, there were no reduced levels or expression of any synovial biomarker. Therefore, the results do not support the view that CCR2 antagonists are sufficient to induce clinical improvement in rheumatoid arthritis, and these studies were discontinued [41]. Moreover, CCR2 and CCR5 antagonists may be able to reduce COVID-19-associated respiratory and cardiovascular organ failure by inhibiting the CCR2 and CCR5 pathways, which could reduce or prevent inflammation or fibrosis in early and late stages of the disease and improve its outcomes, and is currently investigated in a clinical trial [42]. In addition, clinical trials on CCR2 inhibition have focused on diabetes [43], multiple sclerosis [44], atherosclerosis [45], and childhood interstitial pneumonia [46]. Currently, there are only a few studies on CCR2 and SAH. PD-L1 has been reported to prevent cerebral vasospasm by inhibiting the entry of activated Ly6c^+^ and CCR2^+^ monocytes into the brain [39]. Using *Cx3Cr1Gfp*/^+^*Ccr2Rfp*/^+^ transgenic mice, reactive immune cells were found to be predominantly derived from resident microglial libraries rather than infiltrating macrophages [38]. Our study indicated that CCR2 was highly expressed in neurons after SAH and was involved in neuroinflammation and neuronal apoptosis. The CCR2 antagonist RS could effectively reverse this process.

The PI3K/AKT signaling pathway is a very important signaling pathway involved post-SAH and plays a role in regulating inflammation [20, 23] and apoptosis [30, 47]. A recent study showed that CCR2 can activate the PI3K/AKT signaling pathway, suggesting that the PI3K/AKT signaling pathway is downstream of CCR2 action [48]. In this study, our results showed that inhibiting CCR2 significantly upregulated the expression of p-PI3K, p-AKT, and BCL-2 and downregulated the expression of BAX, CC3, TNF-α, and IL-1β, thereby improving neurological deficits after SAH. These effects of CCR2 were reversed by the PI3K-specific inhibitor LY, which inhibited PI3K and AKT phosphorylation and activated downstream inflammatory and apoptotic molecules. Taken together, our results suggest that activation of the PI3K/AKT signaling pathway underlies CCR2-mediated neuroprotection after SAH.

Finally, we collected CSF from patients with clinical aSAH and healthy controls and detected CCR2, inflammatory factors (IL-1β and TNF-α), and apoptotic factors (BAX and caspase 3). The levels of CCR2, inflammatory factors (IL-1β and TNF-α), and apoptotic factors (BAX and caspase 3) in the CSF of patients with aSAH were higher than those in healthy controls. Pearson’s correlation analysis showed that CCR2 levels correlated positively with TNF-α and BAX levels. This is partially different from the findings of previous studies [27, 49], which may be due to differences in sample size, CSF collection time and method, ELISA kits used, and so on. However, to our surprise, there was no correlation between CCR2 levels in CSF and CC3, IL-1β levels. We speculated that the patient's underlying diseases other than SAH might affect this result. In addition, we found that the CCR2 level was positively correlated with the mRS score. The ROC curve showed that the level of CCR2 in the CSF after SAH had a moderate diagnostic value.

Our study has some limitations. First, we only used intravenous injection and once-off administration at 1 h after SAH, and we did not evaluate the best route and possible treatment window of CCR2 for SAH. In addition, we only studied the neuroprotective effect of CCR2 inhibition on neuroinflammation and neuronal apoptosis after SAH in vivo and in vitro through the PI3K/AKT signaling pathway. Therefore, we cannot rule out the possibility that other CCR2-mediated effects play a protective role in brain injury after SAH. Future studies should be conducted to explore additional functions and their potential mechanisms of action. Finally, the number of clinical samples was small, and there may have been some bias in the results.

## Conclusion

In conclusion, our results suggest that *CCR2* is a key gene that mediates neuroinflammation and neuronal apoptosis following SAH. CCR2 inhibition can improve nerve injury and reduce neuroinflammation and neuronal apoptosis after SAH in vivo and in vitro. In addition, the neuroprotective effect of CCR2 inhibition is mediated at least partly through the PI3K/AKT signaling pathway. Moreover, the CCR2 level in the CSF of patients with SAH was higher than that in the control group and was positively correlated with the mRS scores of patients at the 6-month follow-up. Therefore, treatment with CCR2 antagonists may represent a promising strategy for treating SAH.

## Supplementary Information

**Additional file 1: Figure S1.** (A) Grouping of animal experiments, mortality and exclusion. (B) Representative images showing successful SAH modeling. (C) SAH grading score of each group. **** *P* < 0.0001 vs sham group. SAH, subarachnoid hemorrhage; Vehicle, PBS containing 10% DMSO; RS504394, CCR2 specific antagonist; LY294002, PI3K specific inhibitor; DMSO, Dimethyl sulfoxide.

**Additional file 2: Figure S2.** (Inhibition of CCR2 by RS improves (A) neurological score and reduces (B) brain edema and (C) EB extravasation at 72 h after SAH.

## Data Availability

All data generated or analyzed during the course of this study are included in the published article and its supplementary documents.

## Abbreviations

aSAH: Aneurysmal subarachnoid hemorrhage
CCR2: C–C Motif Chemokine Receptor 2
BBB: Blood–brain barrier
CSF: Cerebrospinal fluid
CTA: Computed tomography angiography
DEGs: Differentially expressed genes
DSA: Digital subtraction angiography
EBI: Early brain injury
ELISA: Enzyme-linked immunosorbent assay
GEO: Gene Expression Omnibus
GO: Gene Ontology
FC: Fold change
DMSO: Dimethyl sulfoxide
Veh: Vehicle
i.c.v.: Intracerebroventricular
KEGG: Kyoto Encyclopedia of Genes and Genomes
LPS: Lipopolysaccharide
LY: LY294002
PPI: Protein–protein interaction
ROC: Receiver operating characteristic
RS: RS504393
TCA: Trichloroacetic acid
TUNEL: Terminal deoxynucleotide transferase dUTP notched-end labeling
EB: Evans blue
TCA: Trichloroacetic acid
mRS: Modified Rankin Scale
EAE: Experimental autoimmune encephalomyelitis
CC3: Cleaved caspase 3
CST: Cell Signaling Technology
DEME: Dulbecco's modified Eagle medium

## References

[1] Moon K, Levitt MR, Almefty RO, Nakaji P, Albuquerque FC, Zabramski JM, McDougall CG, Spetzler RF (2016). Treatment of ruptured anterior communicating artery aneurysms: equipoise in the endovascular era?. Neurosurgery.

[2] Suzuki H, Nakano F (2018). To improve translational research in subarachnoid hemorrhage. Transl Stroke Res.

[3] Muhammad S, Hänggi D (2021). Inflammation and anti-inflammatory targets after aneurysmal subarachnoid hemorrhage. Int J Mol Sci.

[4] Sekerdag E, Solaroglu I, Gursoy-Ozdemir Y (2018). Cell death mechanisms in stroke and novel molecular and cellular treatment options. Curr Neuropharmacol.

[5] Yang EJ, Choi E, Ko J, Kim D-H, Lee J-S, Kim IS (2012). Differential effect of CCL2 on constitutive neutrophil apoptosis between normal and asthmatic subjects. J Cell Physiol.

[6] Chu HX, Arumugam TV, Gelderblom M, Magnus T, Drummond GR, Sobey CG (2014). Role of CCR2 in inflammatory conditions of the central nervous system. J Cereb Blood Flow Metab.

[7] van der Meer P, Ulrich AM, Gonźalez-Scarano F, Lavi E (2000). Immunohistochemical analysis of CCR2, CCR3, CCR5, and CXCR4 in the human brain: potential mechanisms for HIV dementia. Exp Mol Pathol.

[8] Banisadr G, Gosselin R-D, Mechighel P, Rostène W, Kitabgi P, Mélik Parsadaniantz S (2005). Constitutive neuronal expression of CCR2 chemokine receptor and its colocalization with neurotransmitters in normal rat brain: functional effect of MCP-1/CCL2 on calcium mobilization in primary cultured neurons. J Comp Neurol.

[9] Struthers M, Pasternak A (2010). CCR2 antagonists. Curr Top Med Chem.

[10] Cebinelli GCM, de Lima KA, Silva Castanheira FVE, Hiroki CH, Monteiro VVS, de Lima MHF, Nascimento DCB, Alves Filho JC, Cunha TM (2021). Cunha FdQ: CCR2-deficient mice are protected to sepsis by the disruption of the inflammatory monocytes emigration from the bone marrow. J Leukoc Biol.

[11] Hu S, Hua Y, Keep RF, Feng H, Xi G (2019). Deferoxamine therapy reduces brain hemin accumulation after intracerebral hemorrhage in piglets. Exp Neurol.

[12] Yang D, Tong L, Wang D, Wang Y, Wang X, Bai C (2010). Roles of CC chemokine receptors (CCRs) on lipopolysaccharide-induced acute lung injury. Respir Physiol Neurobiol.

[13] Kitagawa K, Wada T, Furuichi K, Hashimoto H, Ishiwata Y, Asano M, Takeya M, Kuziel WA, Matsushima K, Mukaida N, Yokoyama H (2004). Blockade of CCR2 ameliorates progressive fibrosis in kidney. Am J Pathol.

[14] Love MI, Huber W, Anders S (2014). Moderated estimation of fold change and dispersion for RNA-seq data with DESeq2. Genome Biol.

[15] Szklarczyk D, Gable AL, Lyon D, Junge A, Wyder S, Huerta-Cepas J, Simonovic M, Doncheva NT, Morris JH, Bork P (2019). STRING v11: protein-protein association networks with increased coverage, supporting functional discovery in genome-wide experimental datasets. Nucleic Acids Res.

[16] Chin C-H, Chen S-H, Wu H-H, Ho C-W, Ko M-T, Lin C-Y (2014). cytoHubba: identifying hub objects and sub-networks from complex interactome. BMC Syst Biol.

[17] Warde-Farley D, Donaldson SL, Comes O, Zuberi K, Badrawi R, Chao P, Franz M, Grouios C, Kazi F, Lopes CT (2010). The GeneMANIA prediction server: biological network integration for gene prioritization and predicting gene function. Nucleic Acids Res.

[18] Wu T, Hu E, Xu S, Chen M, Guo P, Dai Z, Feng T, Zhou L, Tang W, Zhan L (2021). clusterProfiler 4.0: a universal enrichment tool for interpreting omics data. Innovation.

[19] Chen J, Zhang C, Yan T, Yang L, Wang Y, Shi Z, Li M, Chen Q (2021). Atorvastatin ameliorates early brain injury after subarachnoid hemorrhage via inhibition of pyroptosis and neuroinflammation. J Cell Physiol.

[20] Zhu Q, Enkhjargal B, Huang L, Zhang T, Sun C, Xie Z, Wu P, Mo J, Tang J, Xie Z, Zhang JH (2018). Aggf1 attenuates neuroinflammation and BBB disruption via PI3K/Akt/NF-κB pathway after subarachnoid hemorrhage in rats. J Neuroinflammation.

[21] Sugawara T, Ayer R, Jadhav V, Zhang JH (2008). A new grading system evaluating bleeding scale in filament perforation subarachnoid hemorrhage rat model. J Neurosci Methods.

[22] Peng J, Pang J, Huang L, Enkhjargal B, Zhang T, Mo J, Wu P, Xu W, Zuo Y, Peng J (2019). LRP1 activation attenuates white matter injury by modulating microglial polarization through Shc1/PI3K/Akt pathway after subarachnoid hemorrhage in rats. Redox Biol.

[23] Tian Y, Liu B, Li Y, Zhang Y, Shao J, Wu P, Xu C, Chen G, Shi H (2022). Activation of RARα receptor attenuates neuroinflammation after SAH promoting M1-to-M2 phenotypic polarization of microglia and regulating Mafb/Msr1/PI3K-Akt/NF-κB pathway. Front Immunol.

[24] Gong P, Zou Y, Zhang W, Tian Q, Han S, Xu Z, Chen Q, Wang X, Li M (2021). The neuroprotective effects of insulin-like growth factor 1 via the Hippo/YAP signaling pathway are mediated by the PI3K/AKT cascade following cerebral ischemia/reperfusion injury. Brain Res Bull.

[25] Cao Y, Li Y, He C, Yan F, Li J-R, Xu H-Z, Zhuang J-F, Zhou H, Peng Y-C, Fu X-J (2021). Selective ferroptosis inhibitor liproxstatin-1 attenuates neurological deficits and neuroinflammation after subarachnoid hemorrhage. Neurosci Bull.

[26] Gong P, Zhang W, Zou C, Han S, Tian Q, Wang J, He P, Guo Y, Li M (2022). Andrographolide attenuates blood-brain barrier disruption, neuronal apoptosis, and oxidative stress through activation of Nrf2/HO-1 signaling pathway in subarachnoid hemorrhage. Neurotox Res.

[27] Lv S-Y, Wu Q, Liu J-P, Shao J, Wen L-L, Xue J, Zhang X-S, Zhang Q-R, Zhang X (2018). Levels of interleukin-1β, interleukin-18, and tumor necrosis factor-α in cerebrospinal fluid of aneurysmal subarachnoid hemorrhage patients may be predictors of early brain injury and clinical prognosis. World Neurosurg.

[28] Lucke-Wold BP, Logsdon AF, Manoranjan B, Turner RC, McConnell E, Vates GE, Huber JD, Rosen CL, Simard JM (2016). Aneurysmal subarachnoid hemorrhage and neuroinflammation: a comprehensive review. Int J Mol Sci.

[29] Zeyu Z, Yuanjian F, Cameron L, Sheng C (2021). The role of immune inflammation in aneurysmal subarachnoid hemorrhage. Exp Neurol.

[30] Wu L-Y, Enkhjargal B, Xie Z-Y, Travis ZD, Sun C-M, Zhou K-R, Zhang T-Y, Zhu Q-Q, Hang C-H, Zhang JH (2020). Recombinant OX40 attenuates neuronal apoptosis through OX40-OX40L/PI3K/AKT signaling pathway following subarachnoid hemorrhage in rats. Exp Neurol.

[31] Tian Q, Liu S, Han S-M, Zhang W, Qin X-Y, Chen J-H, Liu C-L, Guo Y-J, Li M-C (2023). The mechanism and relevant mediators associated with neuronal apoptosis and potential therapeutic targets in subarachnoid hemorrhage. Neural Regen Res.

[32] Fantuzzi L, Borghi P, Ciolli V, Pavlakis G, Belardelli F, Gessani S (1999). Loss of CCR2 expression and functional response to monocyte chemotactic protein (MCP-1) during the differentiation of human monocytes: role of secreted MCP-1 in the regulation of the chemotactic response. Blood.

[33] Tan X, Hu L, Shu Z, Chen L, Li X, Du M, Sun D, Mao X, Deng S, Huang K, Zhang F (2019). Role of CCR2 in the development of streptozotocin-treated diabetic cardiomyopathy. Diabetes.

[34] Dimitrijevic OB, Stamatovic SM, Keep RF, Andjelkovic AV (2007). Absence of the chemokine receptor CCR2 protects against cerebral ischemia/reperfusion injury in mice. Stroke.

[35] Bruban J, Maoui A, Chalour N, An N, Jonet L, Feumi C, Tréton J, Sennlaub F, Behar-Cohen F, Mascarelli F, Dinet V (2011). CCR2/CCL2-mediated inflammation protects photoreceptor cells from amyloid-β-induced apoptosis. Neurobiol Dis.

[36] Zhang K, Wang H, Xu M, Frank JA, Luo J (2018). Role of MCP-1 and CCR2 in ethanol-induced neuroinflammation and neurodegeneration in the developing brain. J Neuroinflamm.

[37] Xu Z, Shi W-H, Xu L-B, Shao M-F, Chen Z-P, Zhu G-C, Hou Q (2019). Resident microglia activate before peripheral monocyte infiltration and p75NTR blockade reduces microglial activation and early brain injury after subarachnoid hemorrhage. ACS Chem Neurosci.

[38] Zheng ZV, Lyu H, Lam SYE, Lam PK, Poon WS, Wong GKC (2020). The dynamics of microglial polarization reveal the resident neuroinflammatory responses after subarachnoid hemorrhage. Transl Stroke Res.

[39] Jackson CM, Choi J, Routkevitch D, Pant A, Saleh L, Ye X, Caplan JM, Huang J, McDougall CG, Pardoll DM (2021). PD-1+ monocytes mediate cerebral vasospasm following subarachnoid hemorrhage. Neurosurgery.

[40] Zimmermann HW, Sterzer V, Sahin H (2014). CCR1 and CCR2 antagonists. Curr Top Med Chem.

[41] Vergunst CE, Gerlag DM, Lopatinskaya L, Klareskog L, Smith MD, van den Bosch F, Dinant HJ, Lee Y, Wyant T, Jacobson EW (2008). Modulation of CCR2 in rheumatoid arthritis: a double-blind, randomized, placebo-controlled clinical trial. Arthr Rheum.

[42] Files DC, Tacke F, O'Sullivan A, Dorr P, Ferguson WG, Powderly WG (2022). Rationale of using the dual chemokine receptor CCR2/CCR5 inhibitor cenicriviroc for the treatment of COVID-19. PLoS Pathog.

[43] Di Prospero NA, Artis E, Andrade-Gordon P, Johnson DL, Vaccaro N, Xi L, Rothenberg P (2014). CCR2 antagonism in patients with type 2 diabetes mellitus: a randomized, placebo-controlled study. Diabetes Obes Metab.

[44] Brodmerkel CM, Huber R, Covington M, Diamond S, Hall L, Collins R, Leffet L, Gallagher K, Feldman P, Collier P (2005). Discovery and pharmacological characterization of a novel rodent-active CCR2 antagonist, INCB3344. J Immunol.

[45] Gilbert J, Lekstrom-Himes J, Donaldson D, Lee Y, Hu M, Xu J, Wyant T, Davidson M (2011). Effect of CC chemokine receptor 2 CCR2 blockade on serum C-reactive protein in individuals at atherosclerotic risk and with a single nucleotide polymorphism of the monocyte chemoattractant protein-1 promoter region. Am J Cardiol.

[46] Hartl D, Griese M, Nicolai T, Zissel G, Prell C, Reinhardt D, Schendel DJ, Krauss-Etschmann S (2005). A role for MCP-1/CCR2 in interstitial lung disease in children. Respir Res.

[47] Okada T, Enkhjargal B, Travis ZD, Ocak U, Tang J, Suzuki H, Zhang JH (2019). FGF-2 attenuates neuronal apoptosis via FGFR3/PI3k/Akt signaling pathway after subarachnoid hemorrhage. Mol Neurobiol.

[48] Zhu S, Liu M, Bennett S, Wang Z, Pfleger KDG, Xu J (2021). The molecular structure and role of CCL2 (MCP-1) and C-C chemokine receptor CCR2 in skeletal biology and diseases. J Cell Physiol.

[49] Jiang W, Jin P, Wei W, Jiang W (2020). Apoptosis in cerebrospinal fluid as outcome predictors in severe traumatic brain injury: an observational study. Medicine.

