# Supplementary figures and images for: Inhibition of CCR2 attenuates neuroinflammation and neuronal apoptosis after subarachnoid hemorrhage through the PI3K/Akt pathway
Source: J Neuroinflammation. 2022 Dec 25;19:312. doi: 10.1186/s12974-022-02676-8 (PMC9789920; doi:10.1186/s12974-022-02676-8)

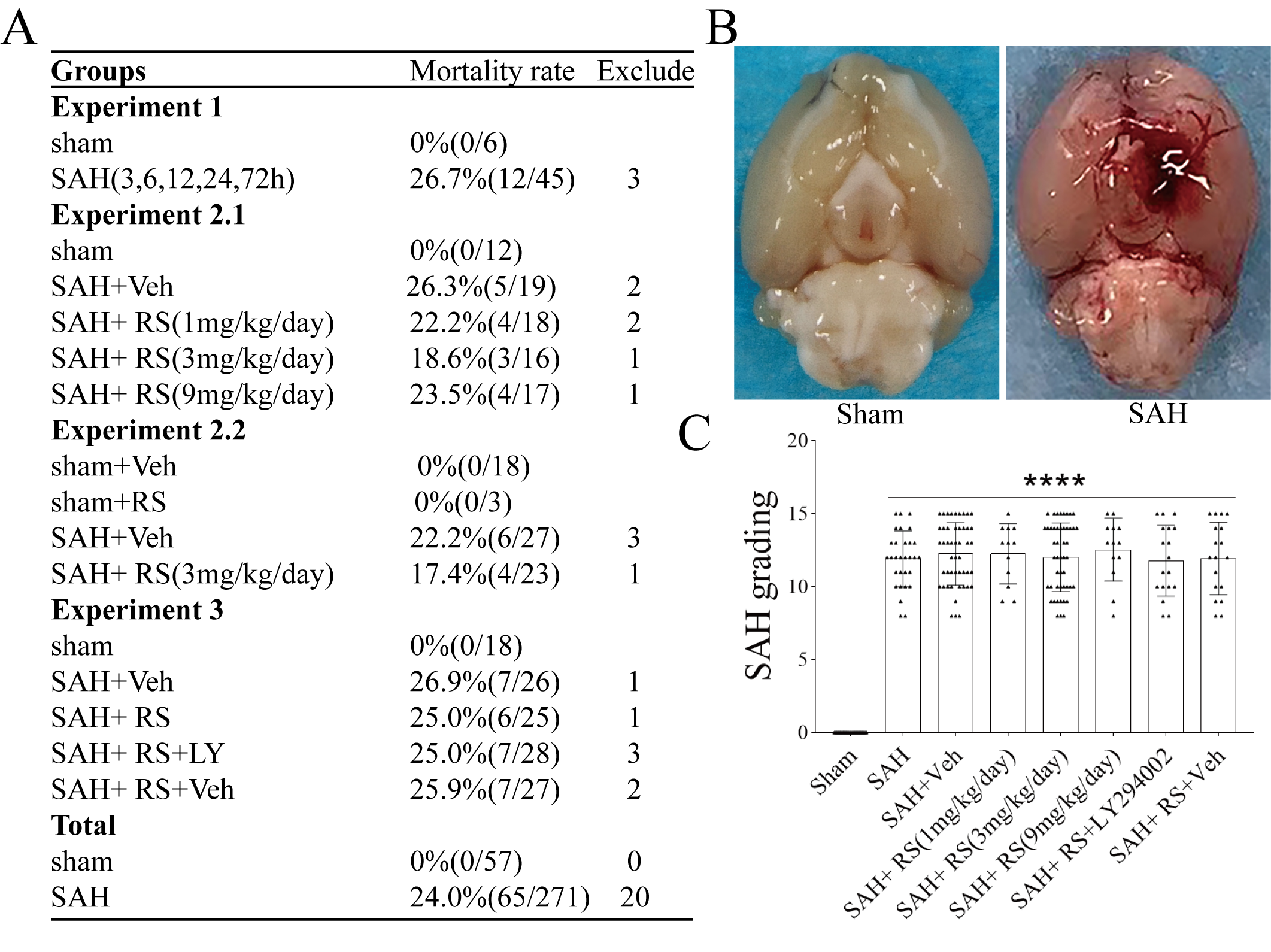

Supplement: Supplementary file 1 — Additional file 1: Figure S1. (A) Grouping of animal experiments, mortality and exclusion. (B) Representative images showing successful SAH modeling. (C) SAH grading score of each group. **** P < 0.0001 vs sham group. SAH, subarachnoid hemorrhage; Vehicle, PBS containing 10% DMSO; RS504394, CCR2 specific antagonist; LY294002, PI3K specific inhibitor; DMSO, Dimethyl sulfoxide. [file 12974_2022_2676_MOESM1_ESM.tif]

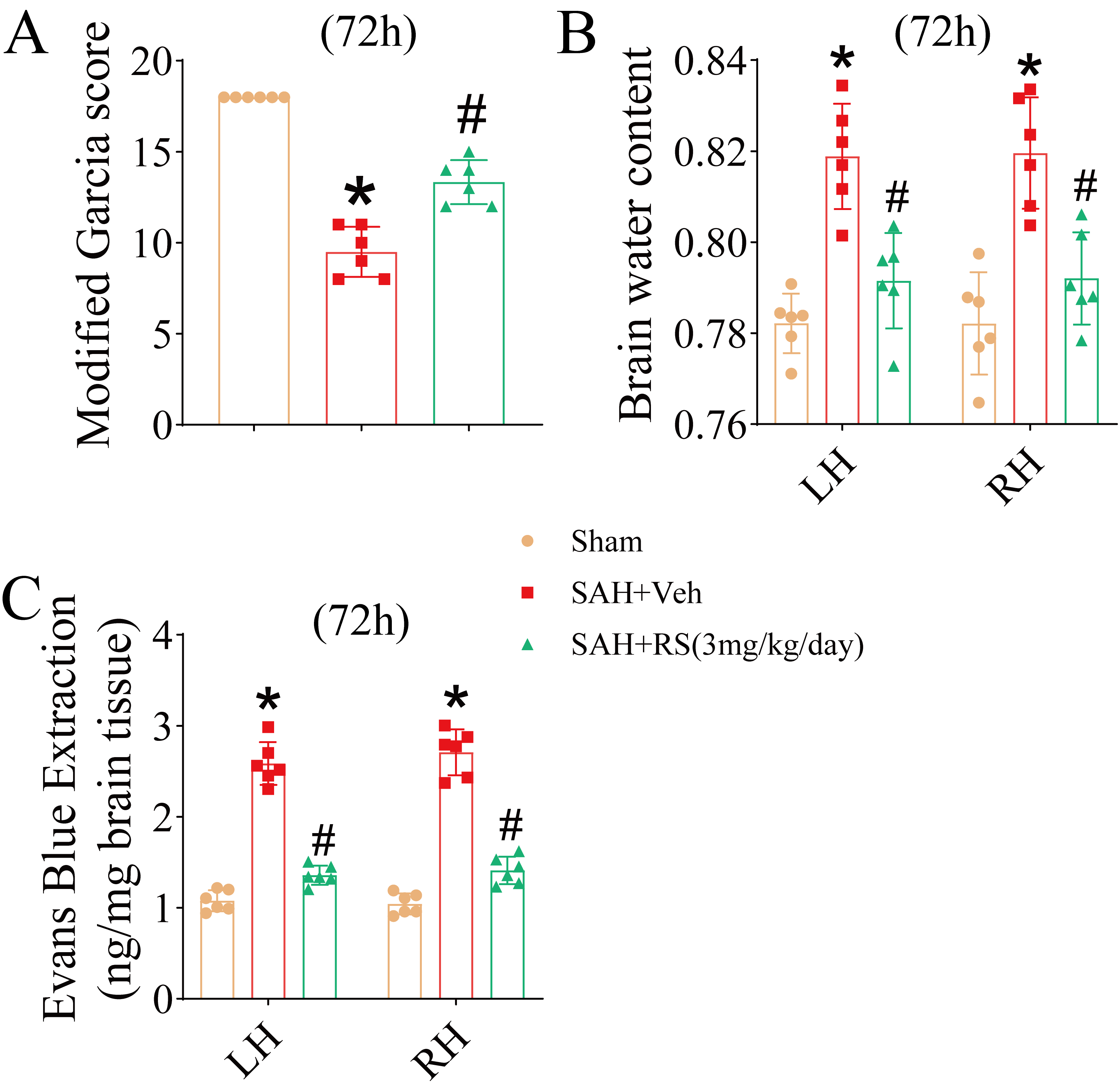

Supplement: Supplementary file 2 — Additional file 2: Figure S2. (Inhibition of CCR2 by RS improves (A) neurological score and reduces (B) brain edema and (C) EB extravasation at 72 h after SAH. [file 12974_2022_2676_MOESM2_ESM.tif]
